# Fourth Annual Meeting of the Mississippi Valley Association of Dental Surgeons

**Published:** 1848-10

**Authors:** 


*Fourth Annual Meeting of the Mississippi Valley Association of Dental Surgeons.*—This Society met pursuant to adjournment, in the lecture room of the Ohio College of Dental Surgery, on Tuesday, Sept. 5th, 1848, at 10 o'clock, A. M.

On calling the roll, the following members answered to their names viz. Dr. Wm. B Ross, President, Drs. Edward Taylor, A. Berry, C. Bonsall, W. M. Hunter, A. N. Newton, D. B. Wheeler, and Jas. Taylor.

The Recording Secretary being absent on account of illness, Dr. C. Bonsall was appointed Secretary, pro tem.

On motion of Dr. E. Taylor, the President filled the vacancies in the Examining and Executive Committees, and when filled, stood as follows:

*Examining Committee*—Drs. E. Taylor, Hunter and Newton.

*Executive Committee*—Drs. A. Berry, Bonsall, and Wheeler.

The Examining Committee reported the following gentlemen for membership:

Dr. B. F. Whitney, of Clarksville, Tenn.
Dr. Jonathan Taft, of Xenia, Ohio.
Dr. A. M. Leslie, of Cincinnati, Ohio.

Who upon being balloted for, were duly elected.

On motion of Dr. E. Taylor, the eleventh article of the constitution was amended, by an unanimous vote, so as to require five, instead of seven, members, besides the President, to constitute a quorum, at the opening of any annual meeting.

The Treasurer presented his account, showing a balance in the treasury of $17 60, which report was referred to an auditing committee, consisting of Drs. Berry and Leslie.

On motion, adjourned to meet at the office of Drs. J. & E. Taylor, this evening, at 7½ o'clock.

Previous to the adjournment, the President gave notice to the Society, that he was about retiring from the profession, briefly, but feelingly thanked the Society for their honor and kindness, and remarked that he should always look to the doings of the Society, with much interest, and rejoice in the good it may accomplish; yet, having embarked in another pursuit, he felt it his duty to resign.

With much regret on the part of the Society, this resignation was accepted.

*Evening Session, 7½ o'clock.*

The Society met according to adjournment, and Dr. Berry was appointed President, pro tem.

Minutes were read and approved.

On motion of Dr. E. Taylor, it was unanimously resolved that the Editors of the Register be exempt from the payment of their annual dues.

The Examining Committee reported the names of the following gentlemen for membership:

Dr. J. G. Curtis, of Hillsboro, Ohio.
Dr. G. L. Vaneman, Cincinnati, Ohio.
Dr. H. McCullum, Augusta, Ky.

Dr. R. E. Cole, New Orleans, La.
Dr. H. D. Stratton, St. Louis, Mo.

Who on being balloted for, were duly elected.

On motion, Dr. J. M. Brown, of Cincinnati, was elected an honorary member.

The Auditing Committee handed in the following report:

*To the Mississippi Valley Association of Dental Surgeons:*

Your Auditing Committee beg leave to report that they have examined the Treasurer's account, and find it correct.

All of which is respectfully submitted,

A. BERRY,

A. M. LESLIE, } *Committee.*

*Cincinnati,* Sept. 5th, 1848.

Report accepted.

The Corresponding Secretary handed in his report.

The Secretary's Report was, on motion, accepted, and the editors of the Register authorized to appoint such agents as they may think proper, to procure subscriptions to the work, on the usual terms.

The address of Dr. B. B. Brown, of St. Louis, on Hemorrhage, now being called for—was by the Secretary read, accepted by the Society—and after eliciting considerable discussion on the general subject of hemorrhage, was ordered to be published in the Register.

On motion, adjourned to meet to-morrow, at 9 o'clock, A. M. in the lecture room of the Ohio College of Dental Surgery.

*Wednesday, Sept. 6th, 9 o'clock, A. M.*

The Society met pursuant to adjournment.

Drs. Goddard and Griffith, of Louisville, having arrived, took their seats with the Society, and in the absence of the President, Dr. W. H. Goddard was elected President, pro tem.

The minutes of the evening session, were read, approved, and accepted.

Dr. Bonsall being now called on for his essay on *Pivot Teeth*, read a brief, but interesting, paper, on that subject, which after being accepted, elicited considerable discussion, in which all the members present joined, making altogether, one of the most instructive and interesting sessions ever held by the Society.

The Committee, appointed at a previous meeting of the Society, to answer interrogatories propounded by Dr. Drake, in relation to the teeth, and their diseases, was, by request, continued.

The interrogatory in relation to decay of the teeth of the blacks, was brought before the Society, and the observations of Drs. Whitney, Berry, Griffith, J. and E. Taylor, and others who had operated much in the south, was made known on this subject, so that the committee will be enabled to give a more satisfactory answer, in relation to this question.

On motion of Dr. E. Taylor the following resolutions were passed :

1st. *Resolved,* That this Society offer to the manufacturer a gold medal, worth twenty dollars, for one hundred of the best teeth offered; including specimens of gum teeth, of molars, and bicuspid, and common plate and pivot teeth, in equal proportions; and that the second best, of the same amount and character, be presented a silver medal, worth ten dollars.

2d. *Resolved,* That the teeth thus presented, be equally distributed among the members present at the next annual meeting, who may not be in arrears.

3d. *Resolved,* That the Corresponding Secretary be directed to forward to each manufacturer in the United States, whom he can ascertain, the foregoing resolutions.

On motion of Dr. Berry.

*Resolved,* That the second day of our next session, be devoted to the discussion of the best method of filling teeth, and that a committee of three be appointed to report on that subject, and also on general practice.

Drs. Berry, James Taylor, and Whitney, were appointed said committee.

On motion of Dr. Bonsall, the Society went into an election of officers for the ensuing year—which resulted as follows:

W. H. Goddard, *President*
Samuel Griffith, 1*st* *Vice-prest.*
B. T. Whitney, 2*d* "
R. E. Cole, 3*d* "
John Allen, *Rec. Secretary.*
Edward Taylor, *Cor.* "
Charles Bonsall, *Treasurer.*

Jas. Taylor, J. Taft, A. Berry, } *Examining Committee.*

B. T. Whitney, S. Griffith, J. G. Curtis, } *Executive Committee.*

Jas. Taylor, B. B. Brown, W. H. Goddard, } *Editing and Pub. Committee.*

The following gentlemen were appointed to deliver addresses at the next annual meeting—the President the opening address:

Dr. Whitney, on Professional Etiquette; Dr. Cole, on Plate Teeth; Dr. Leslie, on Metallurgy; Dr. Curtis, on Filing Teeth; Dr. Taft, on Extracting Teeth; Dr. Griffith, on Alveolar Abscess; Dr. Berry, on Effects of different kinds of food on the Teeth; Dr. Joseph Taylor, on Diseases of the Gums.

On motion of Dr. Bonsall, the thanks of the Association were tendered to the Trustees and Faculty of the Ohio Collge of Dental Surgery, for the use of their hall.

On motion of Dr. Berry, members were requested to bring in any improved instruments they may obtain, to exhibit at the next annual meeting.

On motion of Dr. Leslie, the thanks of the Society were tendered to Drs. Taylor and Brown, for the faithful performance of their duties as editors of the Register.

On motion of Dr. Whitney, members were requested to bring in any interesting morbid specimens they may have, to our next meeting.

On motion of Dr. E. Taylor, the first evening of our next meeting be appropriated for the purpose of an interlocutory session, with special reference to any improvements, which may have become known in dental science.

The Corresponding Secretary was directed to write to each member who is in arrears, informing him, if unpaid at the time of issuing the next No. of the Register, their names will be stricken off the list of members, which will be published at that time.

Ordered that these proceedings be published in the first number of 2d volume of Dental Register.

Minutes were read and approved, and the Society adjourned, to meet on the second Tuesday of September, 1849, at 10 o'clock, A. M. in Louisville, Ky. [*Dental Register.*

